# Activation Energy Impact on Flow of AA7072-AA7075/Water-Based Hybrid Nanofluid through a Cone, Wedge and Plate

**DOI:** 10.3390/mi13020302

**Published:** 2022-02-16

**Authors:** Maaliger B. Rekha, Ioannis E. Sarris, Javali K. Madhukesh, Kondethimmanahalli R. Raghunatha, Ballajja C. Prasannakumara

**Affiliations:** 1 Department of Studies in Mathematics, Davangere University, Davangere 577002, India; mbrekha93@gmail.com (M.B.R.); madhukeshjk@gmail.com (J.K.M.); raghunatha13@gmail.com (K.R.R.); prasannakumarabc@davangereuniversity.ac.in (B.C.P.); 2 Department of Mechanical Engineering, University of West Attica, 12244 Athens, Greece

**Keywords:** hybrid nanofluid, porous medium, heat source/sink, cone, wedge and plate, activation energy

## Abstract

The present research investigates the effect of a heat source/sink on nanofluid flow through a cone, wedge, and plate when using a suspension of aluminium alloys (AA7072 and AA7075) as nanoparticles in base fluid water. The activation energy and porous material are also considered in the modelling. Using similarity transformations, the modelling equations were converted into an ordinary differential equation (ODEs) system. The Runge Kutta Fehlberg 45 fourth fifth-order (RKF 45) technique and shooting approach were used to numerically solve these equations. The influence of essential aspects on flow fields, heat, and mass transfer rates was studied and addressed using graphical representations. The outcome reveals that the case of fluid flow past a plate shows improved heat transfer for augmented heat source/sink parameter values than the cases for fluid flow past a cone and wedge does. Furthermore, we observed the least heat transfer for the case of fluid flow past the cone. The mass transfer for the case of fluid flow past the cone increased more slowly for growing activation energy parameter values than in the other cases. Moreover, we observed higher mass transfer rates for the case of fluid flow past the plate. The augmented values of the heat source/sink parameter decayed the heat transfer rate in all three flow cases.

## 1. Introduction

Nanofluids are heat transfer liquids that include nanoparticle (1–100 nm) suspensions that are dispersed throughout liquid. Water, organic fluids, motor oil, bio-liquids, and other basic liquids are usually included in base liquids. Choi was the first to investigate thermal conductivity augmentation in nanofluids. Recently, many studies have been conducted using two kinds of nanoparticles suspended in a base liquid known as a hybrid nanofluid. The main advantage of using a hybrid nanofluid is that by selecting the right mix of nanoparticles, favourable characteristics may be enhanced, and drawbacks can be mitigated due to their synergistic impact. These hybrid nanofluids are a relatively new type of nanofluids and have a wide range of heat transfer applications, including in microfluidics, transport, medical, defence, naval construction, and acoustics applications. Hybrid nanoparticles provide a colossal advantage and have remarkably high effective heat conductivity when the nano-sized particles are spread correctly. In particular, nanofluid flow is well-known for its high heat transfer compared to conventional fluid. Hybrid nanofluids are utilized to augment this heat transfer ability even further. Recently, Gul [1] analyzed thermal management in hybrid nanofluid flow within a narrowed gap among a disk and cone. Abbas et al. [2] investigated the stagnation point stream of a hybrid nanofluid over a moving cylinder with a magnetic field. Madhukesh et al. [3] swotted alloy–water-based hybrid nanoliquid flow past a coiled sheet with Newtonian heating. Gowda et al. [4] swotted carbon nanotube–water-based hybrid nanoliquid flow past a poignant spinning disk. Mabood et al. [5] educed radiative melting heat transport in copper–alumina–water-based hybrid nanoliquid flow.

Due to its vast variety of applications in science and technology, researchers have been paying close attention to liquid flow through various geometries, such as a vertical cone, wedge, and plate geometries, among others. Many scientists and researchers have looked at this issue from various angles. Waini et al. [6] investigated heat transfer through a hybrid nanofluid using a wedge. Maleki et al. [7] investigated nanofluid flow heat transfer via a moving porous plate. Anwar et al. [8] used a plate embedded in porous media to study the impact of radiation on a second-grade liquid. Kumar et al. [9] looked at how heat radiation affected MHD nanofluid flow across an upright plate. Khan et al. [10] studied the entropic generation of a viscous MHD fluid stream flowing through a spinning cone. Maxwell hybrid nanofluid flow over an upright cone with Cattaneo–Christov heat flux was investigated by Reddy et al. [11]. The effect of non-Fourier heat flux on dusty liquid flow across a plate, cone, and wedge was explained by Mahanthesh et al. [12]. The effect of suction/injection on Williamson liquid flow via a cone and wedge was elucidated by Dawar et al. [13]. The influence of Gyrotactic Microorganisms on Carreau fluid flow via a cone and wedge was explained by Muntazir et al. [14].

A porous medium is a material volume that is made up of a solid matrix and a network of voids. Flows across porous media have a variety of practical uses in nature, including slurries, petroleum reservoir rocks, sand beds, and sedimentation. Furthermore, because of its wide range of practical applications, the study of fluid flow issues in porous media has sparked a lot of interest. Jamshed et al. [15] educed the dissipative stream of a Cu–Al2O3 /engine oil hybrid nanofluid with a porous medium. Gowda et al. [16] quizzed the dual-phase flow of titania–copper/water-based hybrid nanoliquid flow past a cylinder with a porous medium. Kumar et al. [17] conferred the flow of Casson liquid with a dual nanoparticle suspension on a poignant disk with a porous medium. Jawad et al. [18] illuminated the convective Marangoni stream of nanoliquid above a porous surface. Madhukesh et al. [19,20] educed the stream of different liquids past different surfaces in the presence of a porous medium. 

Many researchers have conducted experiments on porous media. Longo et al. [21] verified power-law non-Newtonian axisymmetric porous gravity currents experimentally. They discovered good agreement between theoretical and experimental outcomes in their investigation. Federico et al. [22] investigated radial gravity currents in vertically graded porous media, describing both theory and experiments for Newtonian and power-law fluids. The behaviour of axisymmetric gravity currents of Newtonian and power-law fluids in inhomogeneous porous media is investigated theoretically and empirically in this paper. The obtained results reveal that the rheological characteristics of the invading fluid and changes in permeability have a substantial effect on the radius and profile of the gravity-driven currents that are propagated in porous media. Axisymmetric gravity currents in porous media were experimentally validated by Longo and Federico [23]. Both first-order and zero-order theoretical models were subjected to a sensitivity and uncertainty analysis. The study revealed that porosity fluctuations are more sensitive to the flow process than other parameters are. Some important works highlighting experimental studies in porous media are reported in [24,25,26,27,28].

During cooling processes, heat source/sink (HSS) influence is critical. In most published publications, it is also worth noting that the impact of an interior heat source/sink is usually treated as a temperature-dependent uniform heat source/sink or a space-dependent heat source. All of these studies discovered that the internal heat source mechanism accelerates the formation of the thermal boundary layer. The HSS feature on a nanoliquid stream initiated by a rotating geometry was investigated by Awais et al. [29]. The influence of HSS on nanoliquid flow across a permeable rotating disc was investigated by Nadeem et al. [30]. The effect of HSS on a liquid flow generated by a thin needle was elucidated by Ramzan et al. [31]. Heat transference in nanoliquid over a stretching surface (SS) was defined by Upreti et al. [32] using HSS. Jamaludin et al. [33] investigated the flow pattern of a nanoliquid across an SS using HSS. The activation energy mechanism in hybrid carbon nanotubes and induced magnetic slip flow with a heat source/sink was studied by Ramesh and Madhukesh [34].

Due to its uses in many industrial and mechanical processes, such as fog formation, fibre insulation, air pollution, and catalysis, engineers and scientists have been paying close attention to investigation into the activation energy in binary chemical reactions. It has several practical applications in biochemical systems, combustion, and ceramic manufacture. These applications have prompted a number of researchers to study the influence of these effects on various liquid streams with different geometries. Zaib et al. [35] highlighted the aspects of the radiation effect on a stream Carreau nanoliquid by considering the activation energy. Ijaz et al. [36] debriefed the nanoliquid stream over a revolving disk by considering the activation energy. Ramesh et al. [37] probed the significance of particle movement across parallel plates with activation energy and chemical reaction in aluminium alloys. Bhatti and Michaelides [38] typified the bioconvection flow of nanofluids above a Riga plate. Khan and Nadeem [39] typified the activation energy impact on a Maxwell nanoliquid past an exponentially stretching sheet.

Considering the above-cited articles, we examined the effect of the uniform heat sink/source effect on an incompressible stream of nanofluid across a cone, wedge, and plate in the presence of a porous medium that has not been previously study. Furthermore, activation energy is considered in the modelling. However, to the best of the authors’ knowledge, no numerical solution has been examined for the impact of the heat sink/source effect on the flow of nanoliquid through a cone, wedge, and plate. The main focus of the present paper is on numerically examining the previously stated flow.

## 2. Mathematical Formulation

**Figure 1:**
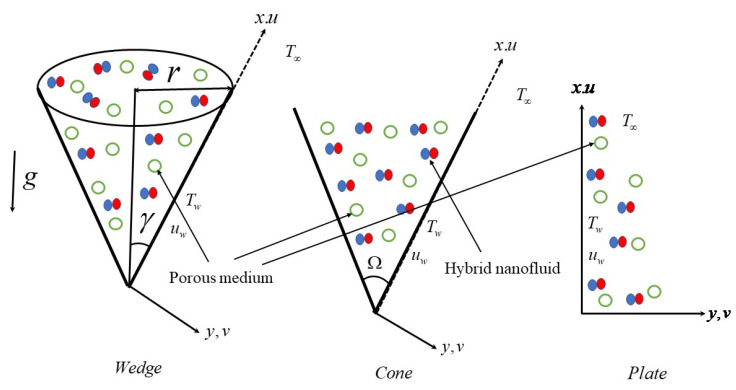
Flow geometry.

Consider a steady, two-dimensional, and incompressible hybrid nanofluid flow over three different geometries (cone, wedge, and plate) in the presence of a porous medium, heat source/sink, and activation energy. The axis x is considered to be along the body’s surface, and y  is normal to its surface. Figure 1 shows the physical model of the model. Let us assume that (γ,Ω,r) are the half-angle of the cone/wedge, full angle of the wedge, and radius of the cone, respectively, and $T_{w}$ is the temperature near the surface (i.e., y=0); furthermore it is assumed that an applied temperature $T_{w}$ routinely warms the novel solid structure, and $T_{\infty }\left(T_{\infty }<T_{w}\right)$ is the far-field temperature (i.e., y→∞), $C_{w}$ is the concentration near the surface (i.e., y=0), and $C_{\infty }$ is the far-field concentration (i.e., y→∞), representing thermodynamic boundary circumstances.

The governing equations for continuity, momentum, temperature, and concentration are below and are based on the assumptions stated above (see Vajravelu and Nayfeh [40], Chetteti and Chukka [41]).

(1) $$\frac{\partial \left(r^{n_{2}}u\right)}{\partial x}+\frac{\partial \left(r^{n_{2}}v\right)}{\partial x}=0,$$

(2) $$u\frac{\partial u}{\partial x}+v\frac{\partial u}{\partial y}+\nu_{hnf}\frac{u}{K^{*}}-\frac{g\rho_{f}\left(T-T_{\infty }\right)\beta_{T}\cos\gamma }{\rho_{hnf}}=\nu_{hnf}\frac{\partial^{2}u}{\partial y^{2}}$$

(3) $$v\frac{\partial T}{\partial y}+u\frac{\partial T}{\partial x}=\frac{k_{hnf}}{{\left(\rho C_{p}\right)}_{hnf}}\frac{\partial^{2}T}{\partial y^{2}}+\frac{Q_{1}}{{\left(\rho C_{p}\right)}_{hnf}}\left(T-T_{\infty }\right)$$

(4) $$v\frac{\partial C}{\partial y}+u\frac{\partial C}{\partial x}=D_{B}\frac{\partial^{2}C}{\partial y^{2}}-k_{r}^{2}{\left(\frac{T}{T_{\infty }}\right)}^{n}e^{\frac{-Ea}{KT}}\left(C-C_{\infty }\right)$$

The boundary conditions are below, (see Ali and Sandeep [42]) and

(5) $$\begin{array}{l} u\to 0,\\ T\to T_{\infty },\\ C\to C_{\infty } \end{array}\}as\;\;y\to \infty$$

In Equations (1)–(5), u,v signifies the velocity components along the x,y directions. g is acceleration due to gravity, $\beta_{T}$ is the volumetric thermal expansion coefficient, $K^{*}$ is the permeability of the porous medium, γ is the half angle of the cone/wedge, $\nu \left(=\frac{\mu }{\rho }\right)$ is the kinematic viscosity of the fluid, ρ is the density of the fluid, μ is the dynamic viscosity of the fluid, $Q_{1}$ is the uniform heat source/sink coefficient, T is the temperature, $T_{w}$ and $T_{\infty }$ denotes temperature near the surface and ambient temperature, respectively, $C_{p}$ is the specific heat at constant pressure, k is the thermal conductivity, $\rho C_{p}$ is the heat capacitance, $k_{r}^{2}$ is the reaction rate, n is the fitted rate constant, Ea is the activation energy, K is the Boltzmann constant, ${\left(\frac{T}{T_{\infty }}\right)}^{n}e^{\frac{-Ea}{KT}}$ is the modified Arrhenius function, C is the concentration, $C_{w}$ is the concentration at the surface, $C_{\infty }$ is the ambient concentration, $D_{B}$ is the Brownian diffusion, l is the characteristic length, and the subscript hnf denotes the hybrid nanoparticles

Based on the following assumptions, three alternative geometries are presented for the suggested problem:

(a) Case 1: Wedge—$n_{2}=0$ and γ≠0;
(b) Case 2: Cone—$n_{2}=1$ and γ≠0;
(c) Case 3: Plate—$n_{2}=0$ and γ=0.

Similarity variables are introduced below:

(6) $$u=\frac{\nu_{f}x}{l^{2}}f^{\prime },v=\frac{-\left(n_{2}+1\right)}{l}f,\eta =\frac{y}{l},\;\theta =\frac{T-T_{\infty }}{T_{w}-T_{\infty }},\chi =\frac{C-C_{\infty }}{C_{w}-C_{\infty }}.$$

From Equation (6), the term u,v represents the velocity components. η denotes the dimensionless similarity variable, and θ and χ represent the dimensionless temperature and concentration profiles, respectively.

The thermophysical properties of the base fluid and nanoparticles are given in Table 1. The expressions for the density, dynamic viscosity, thermal conductivity, and specific heat capacity of hybrid nanofluid are as follows (see Devi and Devi [43]):

$$\rho_{hnf}=\left[\left(1-\phi_{1}\right)\rho_{f}+\phi_{1}\rho_{s_{1}}\right]\left(1-\phi_{2}\right)+\rho_{s_{2}}\phi_{2}\;\mu_{hnf}=\frac{\mu_{f}}{{\left(1-\phi_{1}\right)}^{2.5}{\left(1-\phi_{2}\right)}^{2.5}}\;k_{hnf}=\frac{2k_{nf}+k_{s_{2}}+\left(k_{s_{2}}-k_{nf}\right)2\phi_{2}}{2k_{nf}+k_{s_{2}}-\phi_{2}\left(k_{s_{2}}-k_{nf}\right)}k_{nf}\;\mathrm{and}\;\;k_{nf}=\frac{k_{s_{1}}+2k_{f}-2\phi_{1}\left(k_{f}-k_{s_{1}}\right)}{k_{s_{1}}+2k_{f}+\phi_{1}\left(k_{f}-k_{s_{1}}\right)}k_{f}\;{\left(\rho C_{p}\right)}_{hnf}=\left[\left(1-\phi_{1}\right){\left(\rho C_{p}\right)}_{f}+\phi_{1}{\left(\rho C_{p}\right)}_{s_{1}}\right]\left(1-\phi_{2}\right)+{\left(\rho C_{p}\right)}_{s_{2}}\phi_{2}.$$

In the above expressions, $\phi_{1}$ and $\phi_{2}$ represent the solid volume fractions of AA7075 and AA7072, respectively, and the subscripts $f,\;nf,hnf,s_{1}$ and $s_{2}$ represent the fluid, nanofluid, hybrid nanofluid, and solid particles of AA7075 and AA7072, respectively.

The reduced equations can be expressed as follows

(7) $$\frac{f^{\prime \prime \prime }}{\varsigma_{1}\varsigma_{2}}+f^{\prime \prime }f\left(n_{2}+1\right)-{\left(f^{\prime }\right)}^{2}+\frac{Gr\theta \cos\gamma }{\varsigma_{2}}-\frac{\left(\lambda \right)f^{\prime }}{\varsigma_{1}\varsigma_{2}}=0$$

(8) $$\frac{k_{hnf}}{k_{f}}\frac{\theta^{\prime \prime }}{\mathrm{Pr}\varsigma_{3}}+\left(n_{2}+1\right)f\theta^{\prime }+\frac{Hs\theta }{\varsigma_{3}}=0$$

(9) $$\chi^{\prime \prime }+Sc\left(n_{2}+1\right)f\chi^{\prime }-RcSc{\left(1+\delta \theta \right)}^{n}e^{\frac{-E}{\left(1+\delta \theta \right)}}\chi =0$$

and,

(10) $$\begin{array}{l} \theta \left(0\right)=f^{\prime }\left(0\right)=\chi \left(0\right)=1,\;f\left(0\right)=0\;\;\;\;at\;\;\eta =0\\ \chi \left(\infty \right)=f^{\prime }\left(\infty \right)=\theta \left(\infty \right)=0\;\;\;\;as\;\;\eta \to \infty \; \end{array}\}$$

where $\varsigma_{1}={\left(1-\phi_{1}\right)}^{2.5}{\left(1-\phi_{2}\right)}^{2.5}$, $\varsigma_{2}=\left\{\left(1-\phi_{2}\right)\left[\left(1-\phi_{1}\right)+\phi_{1}\frac{\rho_{s_{1}}}{\rho_{f}}\right]+\phi_{2}\frac{\rho_{s_{2}}}{\rho_{f}}\right\}$ and $\varsigma_{3}=\left(1-\phi_{2}\right)\left[\left(1-\phi_{1}\right)+\phi_{1}\frac{{\left(\rho C_{p}\right)}_{s_{1}}}{{\left(\rho C_{p}\right)}_{f}}\right]+\phi_{2}\frac{{\left(\rho C_{p}\right)}_{s_{2}}}{{\left(\rho C_{p}\right)}_{f}}$

The flow control parameters are as follows:

$Gr=\frac{g\beta \left(T_{w}-T_{\infty }\right)l^{2}}{u_{w}\nu_{f}}$ is the Grashof number, $E=\frac{Ea}{KT_{\infty }}$ is the Activation energy parameter, $\lambda =\frac{l^{2}}{K^{*}}$ is the porosity parameter, $\mathrm{Pr}=\frac{\left(\rho Cp\right)\nu_{f}}{k}$ is the Prandtl number, $Hs=\frac{Q_{1}l^{2}}{{\left(\rho C_{p}\right)}_{f}\nu_{f}}$ is the heat source/sink parameter, $Rc=\frac{k_{r}^{2}l^{2}}{\nu_{f}}$ is the reaction rate parameter, $Sc=\frac{\nu_{f}}{D_{B}}$ is the Schmidt number, and $\delta =\frac{T_{w}-T_{\infty }}{T_{\infty }}$ is the temperature difference.

Using the appropriate similarity transformations, the main physical variables of importance, such as skin friction, Nusselt number, and Sherwood number, are provided below.

(11) $$Cf^{*}=\frac{\tau_{w}}{\rho_{f}u_{w}^{2}},\;Nu=\frac{lq_{w}}{k_{f}\left(T_{w}-T_{\infty }\right)},Sh=\frac{lj_{w}}{D_{B}\left(C_{w}-C_{\infty }\right)}$$

(12) $$\mathrm{Here},\;\tau_{w}=\mu_{hnf}{\frac{\partial u}{\partial y}|}_{y=0},\;q_{w}=-k_{hnf}{\frac{\partial T}{\partial y}|}_{y=0}\;\mathrm{and}\;j_{w}=-D_{B}{\frac{\partial C}{\partial y}|}_{y=0}$$

Substituting Equation (12) into (11), we have

(13) $$Cf=\frac{l}{x}Cf^{*}=\frac{f^{\prime \prime }\left(0\right)}{\varsigma_{1}},\;Nu=\frac{-k_{hnf}}{k_{f}}\theta^{\prime }\left(0\right),\;\mathrm{and}\;Sh=-\chi^{\prime }\left(0\right)\;$$

## 3. Numerical Procedure and Validation

The RKF-45 approach and shooting technique were used to solve the governing Equations (7)–(9) and to reduce the boundary conditions with the aid of well-known computing tools. We transformed the reduced equations into a first-order system.

Let us take

$f=q_{1}$, $f^{\prime }=q_{2}$, $f^{\prime \prime }=q_{3}$, $\theta =q_{4}$, $\theta^{\prime }=q_{5}$, $\chi =q_{6}$ and $\chi^{\prime }=q_{7}$

Through this, we obtain

(14) $$q_{3}^{’}=-\varsigma_{1}\varsigma_{2}\left(q_{3}q_{1}\left(n_{2}+1\right)-{\left(q_{2}\right)}^{2}+\frac{Gr\theta \cos\gamma }{\varsigma_{2}}-\frac{\left(\lambda \right)q_{2}}{\varsigma_{1}\varsigma_{2}}\right)$$

(15) $$q_{5}^{’}=-\mathrm{Pr}\varsigma_{3}\frac{k_{f}}{k_{hnf}}\left(n_{2}+1\right)q_{1}q_{5}+\frac{Hsq_{4}}{\varsigma_{3}}=0$$

(16) $$q_{7}^{’}=RcSc{\left(1+\delta q_{4}\right)}^{n}e^{\frac{-E}{\left(1+\delta q_{4}\right)}}q_{6}-Sc\left(n_{2}+1\right)q_{1}q_{7}$$

Additionally, the boundary conditions become

(17) $$\begin{array}{l} f\left(0\right)=0,\;f^{\prime }\left(0\right)=1,f^{\prime \prime }\left(0\right)=\xi_{1},\\ \theta \left(0\right)=1,\theta^{\prime }\left(0\right)=\xi_{2},\\ \chi \left(0\right)=1\;\&\;\chi^{\prime }\left(0\right)=\xi_{3}.\; \end{array}\}$$

The initial value problem (IVP) in Equations (14)–(16) and (17) is solved numerically with the aid of the RKF-45-order method, and unknown values are attained with the help of a shooting technique by assigning the error tolerance and step size to be $10^{-6}$ and h=0.01 respectively, further gratifying the boundary conditions at the infinity level. The numerical estimations were obtained by utilizing the build-in function of bvp4c in MATLAB by setting the parameters ranges as Gr=1, λ=1, Sc=0.8, Hs=E=n=δ=0.1, and Rc=0.5, Furthermore, the numerical solutions are compared to those from previous studies, resulting in similar findings (see Table 2 and Table 3).

## 4. Results and Discussion

This section explains how to graphically represent the important and pertinent parameters on the involved profiles. With proper plots, the physical quantities of importance, such as skin friction and Nusselt number, are also elucidated. In the current study, we examined fluid flow past three different surfaces: (i) fluid flow past a cone, (ii) fluid flow past a wedge, and (iii) fluid flow past a plate. In the graphs, the dashed curves show flow through a wedge, solid lines indicate flow past a cone, and dotted lines indicate flow past a plate.

**Figure 2:**
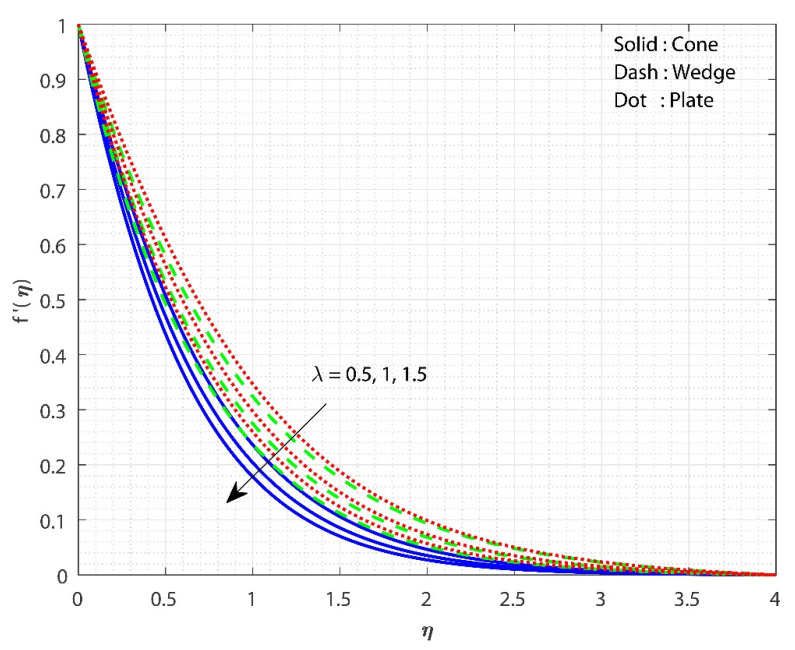
The impact of λ on $f^{\prime }\left(\eta \right)$ when keeping Gr=1, Hs=0.1, Rc=E=0.5, δ=0.1, $n=0.1,\;Sc=0.8,\;\phi_{1}=\phi_{2}=0.01$.

**Figure 3:**
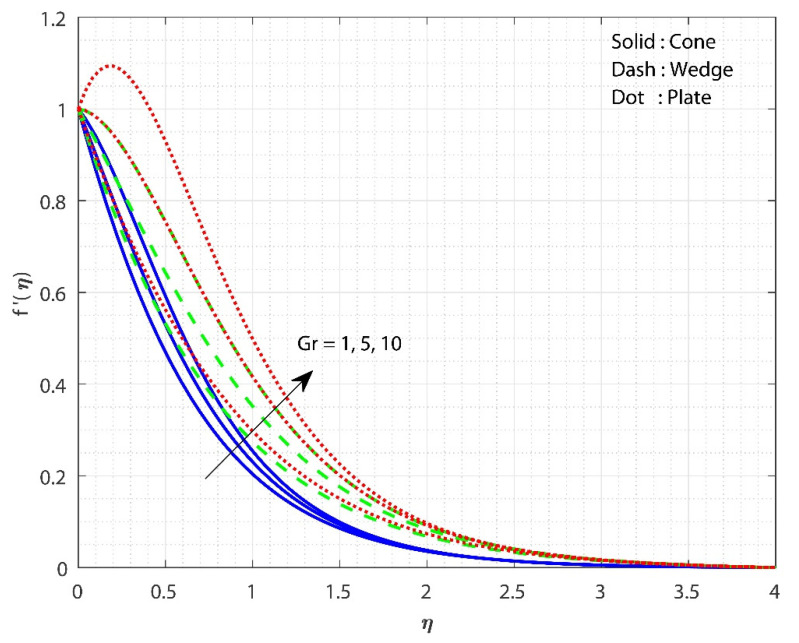
The upshot of Gr on $f^{\prime }\left(\eta \right)$ when keeping λ=1, Hs=0.1, Rc=E=0.5, δ=0.1, $n=0.1,\;Sc=0.8,\;\phi_{1}=\phi_{2}=0.01$.

**Figure 4:**
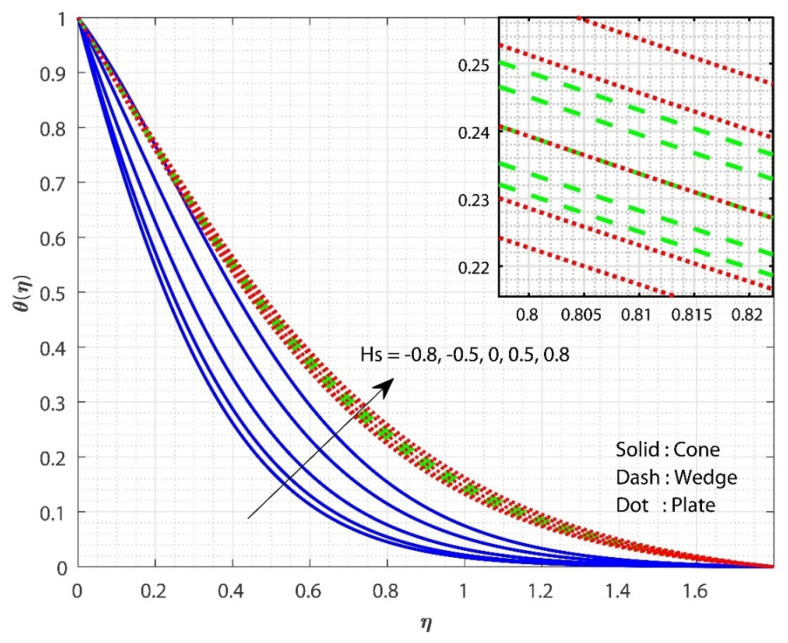
The upshot of Hs on θ(η) when keeping λ=1, Gr=1, Rc=E=0.5, δ=0.1, $n=0.1,\;Sc=0.8,\;\phi_{1}=\phi_{2}=0.01$.

The impact of λ on $f^{\prime }\left(\eta \right)$ is displayed in Figure 2 for three different fluid flow cases. The increase in the λ value declines the $f^{\prime }\left(\eta \right)$ for all three flow cases. As the value of λ rises, so does the system’s resistance. As a significance of the increased frictional force, the fluid flow is reduced. As a result of the additional resistance, the liquid’s velocity decreases. Furthermore, the $f^{\prime }\left(\eta \right)$ for fluid flow past the cone declines faster as the λ values increase than in the remaining cases. The influence of Gr on $f^{\prime }\left(\eta \right)$ is displayed for all the three flow cases in Figure 3. The upsurge in the value of Gr improved the $f^{\prime }\left(\eta \right)$ for all three flow cases. An increase in the Gr values decreases the thickness of the boundary layer due to variations in the buoyancy forces caused by the temperature differences. Furthermore, the $f^{\prime }\left(\eta \right)$ for the fluid flow the past plate inclines faster as the Gr values increase than in the remaining two cases. Figure 4 shows the effect of Hs on θ(η) for all the three flow cases. The upsurge in the Hs values improves the θ(η) for all three flow cases. Moreover, the fluid flow past plate case shows improved heat transfer than the remaining two cases. Here, we observed the least heat transfer for the case of fluid flow past a cone. Internal heat absorption/generation either helps or degrades heat transport. As the Hs grows, the layer θ(η) thickens. The heat source restrictions in the flow state will exhibit better heat transfer. The presence of a heat source energizes the fluid. Consequently, as heat is consumed, the buoyancy force accelerates the flow and improves heat transfer.

**Figure 5:**
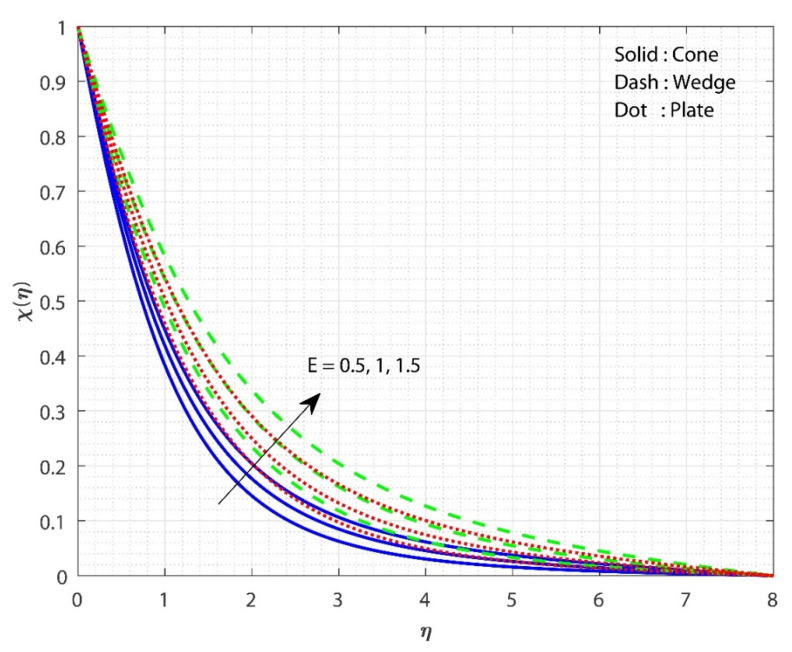
The upshot of E on χ(η) when keeping λ=1, Gr=1, Hs=0.1, Rc=0.5, δ=0.1, $n=0.1,\;Sc=0.8,\;\phi_{1}=\phi_{2}=0.01$.

**Figure 6:**
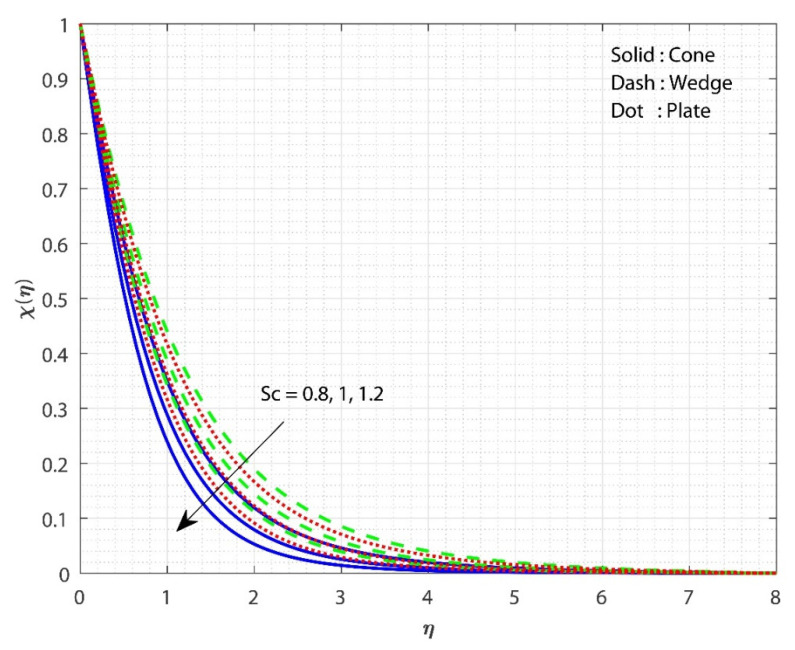
The upshot of Sc on χ(η) when keeping λ=1, Gr=1, Hs=0.1, Rc=E=0.5, δ=0.1, $n=0.1,\;\phi_{1}=\phi_{2}=0.01$.

**Figure 7:**
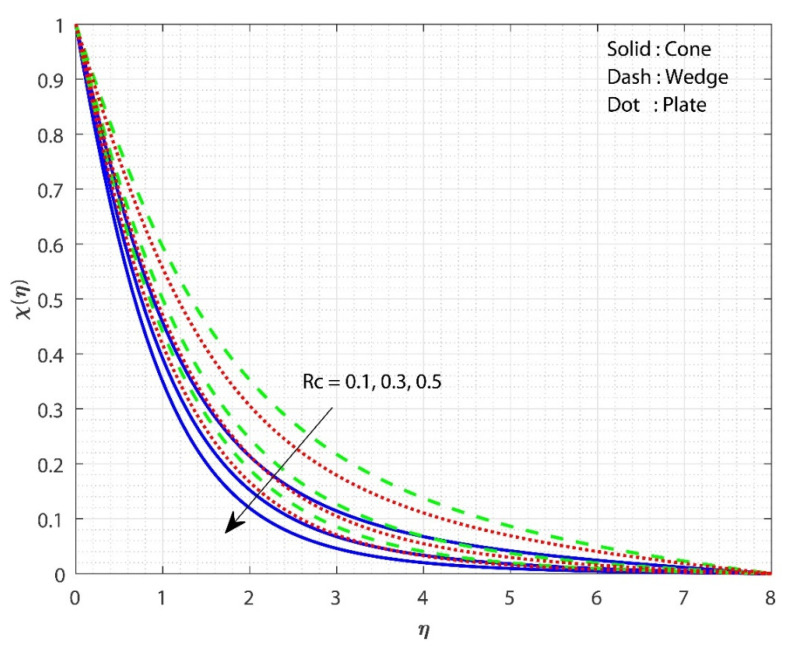
The upshot of Rc on χ(η). when keeping λ=1, Gr=1, Hs=0.1, E=0.5, δ=0.1, $n=0.1\;,Sc=0.8,\;\phi_{1}=\phi_{2}=0.01$.

In Figure 5, the impact of E on χ(η) is displayed for the three different fluid flow cases. The increase in the E value increases the χ(η) for all three flow cases. The impact of the porosity parameter λ on $f^{\prime }\left(\eta \right)$ is the same as the impact of E on χ(η). The Arrhenius equation shows that injecting activation energy into any system causes a reduction in heat and acceleration, resulting in a low response rate constant. As a result, the chemical reaction takes longer to complete, resulting in a larger particle concentration. As E grows, the modified Arrhenius process decays. Consequently, the generative chemical process is accelerated, resulting in an increase in the nanoparticle concentration. As a consequence, the χ(η) increases in value. Furthermore, the χ(η) for fluid flow past the cone increases more slowly as the E value grows than in the remaining cases. Here, we observe higher fluid flow mass transfer when flowing past the plate. Figure 6 shows the effect of Sc on χ(η) for all the three flow cases. The upsurge in the Sc value decreases the χ(η) for all three flow cases. The smallest number indicates the highest concentration of nanoparticles. Momentum diffusivity increases as the Sc rises, causing mass transport to decline. As Sc increases, the diffusion coefficient reduces, lowering the mass transfer. Moreover, the case of fluid flow past the wedge shows better mass transfer than the remaining two cases. Here, we observe the least fluid flow mass transfer when flowing past the wedge. Figure 7 shows the influence of Rc on χ(η) for the three different fluid flow cases. Increasing the Rc value declines the χ(η) for all three flow cases. A larger chemical reaction has a negative impact on the reactant species, degrading them. When Rc increases, the χ(η) is lowered as a result. Furthermore, the χ(η) for the case of fluid flow past a wedge decreases more slowly as the Rc value increases than in the remaining cases. Here, we observe higher mass transfer for the case of fluid flow past a wedge.

**Figure 8:**
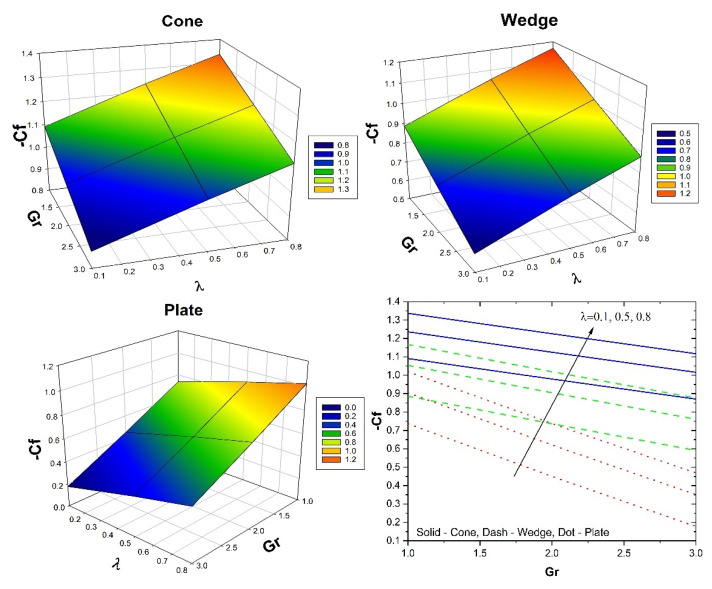
The upshot of λ on skin friction versus Gr for all the three flow cases.

**Figure 9:**
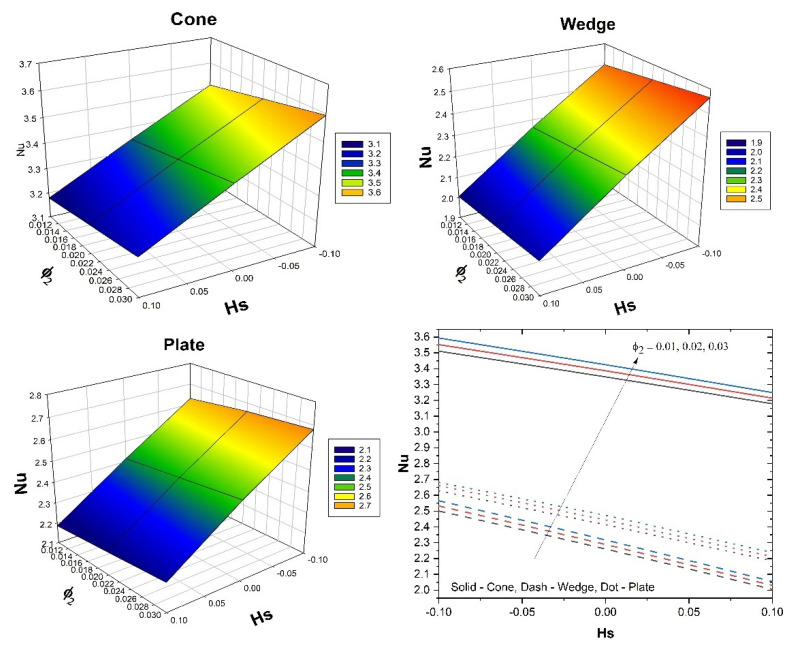
The upshot of $\phi_{2}$ on Nusselt number versus Hs for all the three flow cases.

**Figure 10:**
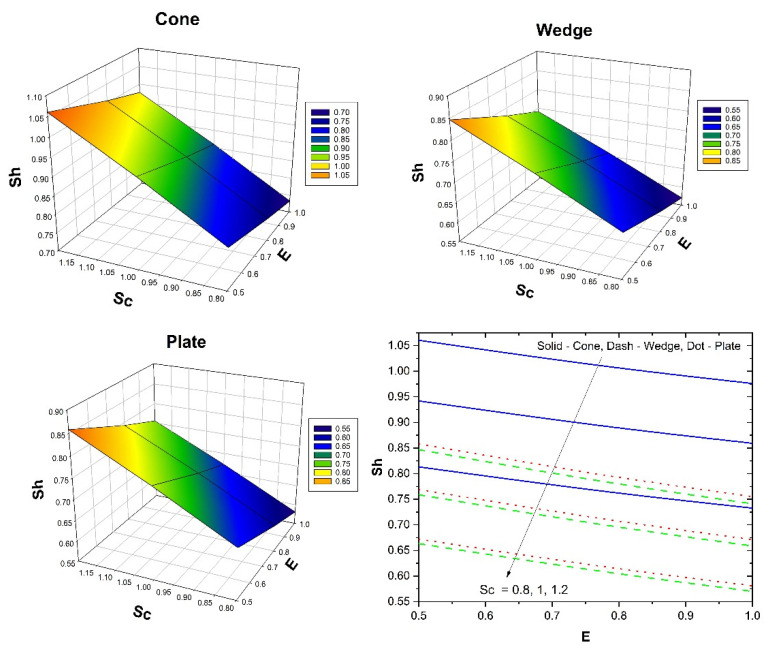
The upshot of Sc on Sherwood number versus E for all the three flow cases.

Figure 8 shows the impact of λ on the skin friction versus the Gr for all the three flow cases. Additionally, the three-dimensional plots in Figure 8 show variation in skin friction for varied values of λ and Gr. Here, the augmented λ values improve the skin friction for all three flow cases, but the inverse behaviour is seen for improved Gr values. Further, the case of fluid flow past the cone shows improved skin friction than the remaining cases. The influence of $\phi_{2}$ on the Nusselt number versus Hs for all the three flow cases is shown in Figure 9. Additionally, the three-dimensional plots in Figure 9 show the variation in the Nusselt number for varied $\phi_{2}$ and Hs values. Here, the augmented $\phi_{2}$ values improve the heat transfer rate for all three flow cases, but the inverse behaviour is seen for improved Hs values. Furthermore, in the case of fluid flow past a cone, an improved heat transfer rate is seen than the remaining cases. The influence of Sc on the Sherwood number versus the E for all the three flow cases is shown in Figure 10. Additionally, the three-dimensional plots in Figure 9 show the variation in the Sherwood number for varied Sc and E values. Here, the augmented E and Sc values decrease the mass transfer rate in all three flow cases. Further, the case of fluid flow past a cone shows improved mass transfer rates than the remaining cases. Table 4 shows the variation in $f^{\prime \prime }\left(0\right)\;\&\;\theta^{\prime }\left(0\right)$ for various parameters in the cone case when Sc=0.8, Kc=Ks=0.1 for different volume fraction combinations. The increase in the Gr values enhances the surface drag force coefficient. From the table, it can be observed that the surface drag force coefficient is higher for the hybrid nanofluid than it is for the nanofluid. This is due to variation in the buoyancy forces caused by the temperature differences. The reverse trend is seen in the case of the coefficient for the rate of thermal distribution. An increase in the λ values reduces the surface drag force coefficient and enhances the rate of thermal distribution. This is due to the presence of the porous medium experiencing the frictional force. It can be seen from the table that in both the cases, the hybrid nanofluid shows better performance than the nanofluid does. An increase in the Hs values increases the surface drag force and thermal distribution coefficients. An increase in the Hs values helps the distribution of heat from the system to the fluid. The Hybrid nanofluid demonstrated better performance than the nanofluid did. Table 5 displays the variation in $f^{\prime \prime }\left(0\right)$ and $\theta^{\prime }\left(0\right)$ for various parameters in the wedge case when Sc=0.8, Kc=Ks=0.1 for different volume fraction combinations. The surface drag force coefficient increases as the Gr values increase. In the case of the coefficient for the rate of thermal dispersion, the opposite tendency is observed. An increase in the λ values decreases the surface drag force coefficient and increases the rate of heat dispersion. Increases in the Hs values increase the surface drag force and heat distribution coefficients. The table shows that in both cases, the hybrid nanofluid has a greater impact than the nanofluid. Table 5 displays the variation in $f^{\prime \prime }\left(0\right)$ and $\theta^{\prime }\left(0\right)$ for various parameters in the plate case when Sc=0.8,Kc=Ks=0.1 for different volume fraction combinations. The surface drag force coefficient increases as the Gr value increases. In the case of the coefficient for the rate of thermal dispersion, the opposite behavior is observed. An increase in the λ values decreases the surface drag force coefficient and increases the pace of heat dispersion. As the Hs values rise, so do the surface drag force and thermal dispersion coefficients. The table demonstrates that hybrid nanofluid has a bigger influence than nanofluid does in both circumstances. From Table 4, Table 5 and Table 6, it is clear that the surface drag force and rate of thermal distribution coefficients have a greater impact in the plate case than in the cone and wedge cases. Table 7 shows the variation in $\chi^{\prime }\left(0\right)$ for various parameters for all three flow cases when Gr=1, λ=1, Hs=0.1, n=0.1 & Sc=0.8. It can be observed form Table 6 that an increase in the E values improves the coefficient for the mass transfer rate. An improvement in the Rc values decreases the coefficient for the mass transfer rate. Increases in the δ values reduce the coefficient for the mass transfer rate. In the case of E, Rc, and δ the influence of the nanofluid is greater than that of the hybrid nanofluid. For all three cases, the wedge shows a better coefficient for mass transfer rate than the other two geometries.

## 5. Conclusions

The present research investigates the effect of heat source/sink on nanofluid flow through a cone, wedge, and plate when a suspension of aluminium alloys (AA7072 and AA7075) is used as nanoparticles in base liquid water. The activation energy and porous material are also considered in the modelling. By employing the appropriate transformations, a set of governing equations was reduced to ODEs, which were then numerically solved using the RKF-45 process using the shooting approach. Graphically, the behaviour of the concentration, temperature, and velocity fields is examined. The main conclusions of the study are listed below:

- The $f^{\prime }\left(\eta \right)$ for the case of fluid flow past cone declines faster for growing λ values than in the remaining two cases.
- The $f^{\prime }\left(\eta \right)$ for the case of fluid flow past the plate increases faster with growing Gr values than in the remaining two cases.
- The case of fluid flow past the plate shows improved heat transfer for augmented Hs values than in the remaining two cases. Further, we observed the least heat transfer in the case of fluid flow past the cone.
- The χ(η) for the case of fluid flow past the cone increases slower with growing E values than in the remaining cases. Moreover, we observed higher mass transfer in the case of fluid flow past the plate.
- The χ(η) for the case of fluid flow past the wedge declines more slowly for growing Rc values than in the remaining cases. Furthermore, we observed higher mass transfer in the case of fluid flow past the wedge.
- Augmented $\phi_{2}$ values improved the heat transfer rate for all three flow cases, but the inverse behaviour was seen for improved Hs values. Further, the case of fluid flow past the cone shows an improved heat transfer rate than the remaining two cases.

## Figures and Tables

**Table 1:** Thermophysical properties of base fluid and nanoparticles. (See Hayat et al. [44], Olatundun and Makinde [45]).

Property	$\rho \left(kg/m^{3}\right)$	$C_{p}\left(J/kgK\right)$	k(W/mK)
Water	997.1	4179	0.613
AA7075	2810	960	173
AA7072	2720	893	222

**Table 2:** Validation of the code for $-\theta^{\prime }\left(0\right)$ diverse values of Pr when $n_{2}=Gr=\lambda =Hs=\phi_{1}=\phi_{2}=0$.

Pr	Hassanien et al. [46]	Salleh et al. [47]	Present Results	CPU Time
0.72	0.46325	0.46317	0.46359	0.845 s
1	0.58198	0.58198	0.58201	0.845 s
3	1.16525	1.16522	1.16525	0.845 s
5	-	1.56806	1.56808	0.845 s
7	-	1.89548	1.89540	0.845 s
10	2.30801	2.30821	2.30801	0.845 s
100	7.74925	7.76249	7.76565	0.845 s

**Table 3:** Computational values of $-f^{\prime \prime }\left(0\right)$ for various values of λ in the absence of $n_{2}=Gr=\phi_{1}=\phi_{2}=0$.

Parameter	Kameswaran et al. [48]		Present Study	CPU Time (In Seconds)
λ	**Analytical**	**Numerical**	**RKF-45**	
0.5	1.22474487	1.22474487	1.224757521	0.034
1	1.41421356	1.41421356	1.414216330	0.034
1.5	1.58113883	1.58113883	1.581138786	0.034
2	1.73205081	1.73205081	1.732050762	0.034
5	2.44948974	2.44948974	2.449489673	0.034

**Table 4:** Variation in $f^{\prime \prime }\left(0\right)\;\&\;\theta^{\prime }\left(0\right)$ for various parameters in the cone when Sc=0.8, E=δ=n=0.1, Rc=0.5.

Parameters			$-f^{\prime \prime }\left(0\right)$		$-\theta^{\prime }\left(0\right)$	
Gr	λ	Hs	$\begin{array}{l} \phi_{1}=0.01\\ \phi_{2}=0.01 \end{array}$	$\begin{array}{l} \phi_{1}=0.01\\ \phi_{2}=0 \end{array}$	$\begin{array}{l} \phi_{1}=0.01\\ \phi_{2}=0.01 \end{array}$	$\begin{array}{l} \phi_{1}=0.01\\ \phi_{2}=0 \end{array}$
1	1	0.1	1.400283	1.410429	1.862376	1.897508
5	1	0.1	0.969792	0.975524	1.932668	1.968334
10	1	0.1	0.465046	0.465352	2.004684	2.041042
1	1	0.1	1.400283	1.410429	1.862376	1.897508
1	1.5	0.1	1.549492	1.562260	1.827821	1.862350
1	2	0.1	1.687038	1.702108	1.795784	1.829781
1	1	−0.5	1.432974	1.442571	2.421712	2.463232
1	1	0	1.423294	1.432838	1.959068	1.995329
1	1	0.5	1.408862	1.418364	1.375103	1.405927

**Table 5:** Variation in $f^{\prime \prime }\left(0\right)\;\&\;\theta^{\prime }\left(0\right)$ for various parameters in the wedge when Sc=0.8, E=δ=n=0.1, Rc=0.5.

Parameters			$-f^{\prime \prime }\left(0\right)$		$-\theta^{\prime }\left(0\right)$	
Gr	λ	Hs	$\begin{array}{l} \phi_{1}=0.01\\ \phi_{2}=0.01 \end{array}$	$\begin{array}{l} \phi_{1}=0.01\\ \phi_{2}=0 \end{array}$	$\begin{array}{l} \phi_{1}=0.01\\ \phi_{2}=0.01 \end{array}$	$\begin{array}{l} \phi_{1}=0.01\\ \phi_{2}=0 \end{array}$
1	1	0.1	1.238079	1.247846	1.180946	1.204269
5	1	0.1	0.675218	0.678814	1.283103	1.307223
10	1	0.1	0.038028	0.034280	1.373024	1.398114
1	1	0.1	1.238079	1.247846	1.180946	1.204269
1	1.5	0.1	1.401688	1.414186	1.138794	1.161443
1	2	0.1	1.550276	1.565133	1.099999	1.122053
1	1	−0.5	1.296383	1.305425	1.962916	1.995014
1	1	0	1.276035	1.284765	1.347111	1.370614
1	1	0.5	1.233634	1.241449	0.359555	0.366689

**Table 6:** Variation in $f^{\prime \prime }\left(0\right)\;\&\;\theta^{\prime }\left(0\right)$ for various parameters in plate when Sc=0.8, E=δ=n=0.1, Rc=0.5.

Parameters			$-f^{\prime \prime }\left(0\right)$		$-\theta^{\prime }\left(0\right)$	
Gr	λ	Hs	$\begin{array}{l} \phi_{1}=0.01\\ \phi_{2}=0.01 \end{array}$	$\begin{array}{l} \phi_{1}=0.01\\ \phi_{2}=0 \end{array}$	$\begin{array}{l} \phi_{1}=0.01\\ \phi_{2}=0.01 \end{array}$	$\begin{array}{l} \phi_{1}=0.01\\ \phi_{2}=0 \end{array}$
1	1	0.1	1.090889	1.099093	1.210755	1.234264
5	1	0.1	0.038028	0.034281	1.373024	1.398114
10	1	0.1	−1.117223	−1.134630	1.501200	1.527898
1	1	0.1	1.090889	1.099093	1.210755	1.234264
1	1.5	0.1	1.256682	1.267646	1.170569	1.193411
1	2	0.1	1.407562	1.420912	1.133601	1.155855
1	1	−0.5	1.175516	1.183083	1.972300	2.004569
1	1	0	1.136729	1.143730	1.367148	1.391100
1	1	0.5	1.060646	1.066209	0.432229	0.441969

**Table 7:** Variation in $\chi^{\prime }\left(0\right)$ for various parameters in three geometries when Gr=1, λ=1, Hs=0.1, n=0.1 & Sc=0.8.

Parameters			Cone $-\chi^{\prime }\left(0\right)$		Wedge $-\chi^{\prime }\left(0\right)$		Plate $-\chi^{\prime }\left(0\right)$	
E	Rc	δ	$\begin{array}{l} \phi_{1}=0.01\\ \phi_{2}=0.01 \end{array}$	$\begin{array}{l} \phi_{1}=0.01\\ \phi_{2}=0 \end{array}$	$\begin{array}{l} \phi_{1}=0.01\\ \phi_{2}=0.01 \end{array}$	$\begin{array}{l} \phi_{1}=0.01\\ \phi_{2}=0 \end{array}$	$\begin{array}{l} \phi_{1}=0.01\\ \phi_{2}=0.01 \end{array}$	$\begin{array}{l} \phi_{1}=0.01\\ \phi_{2}=0 \end{array}$
0.5	0.1	0.1	0.813196	0.895708	0.663420	0.662320	0.672316	0.671157
1.0	0.1	0.1	0.732771	1.101333	0.570258	0.568918	0.580944	0.579525
1.5	0.1	0.1	0.673799	1.267275	0.500316	0.498768	0.512222	0.510573
0.5	0.1	0.1	0.654348	0.652039	0.476496	0.474929	0.486798	0.485121
0.5	0.3	0.1	0.792405	0.790694	0.639072	0.637965	0.647309	0.646135
0.5	0.5	0.1	0.897091	0.895681	0.758106	0.757216	0.765193	0.764258
0.5	0.1	0.1	0.897119	0.895708	0.758126	0.757237	0.765217	0.764282
0.5	0.1	0.2	0.897701	0.896271	0.759113	0.758197	0.766724	0.765765
0.5	0.1	0.3	0.898182	0.896733	0.759966	0.759026	0.768101	0.767120
